# Comparison of the efficiencies of intrathecal and intraganglionic injections in mouse dorsal root ganglion

**DOI:** 10.55730/1300-0144.5702

**Published:** 2023-08-11

**Authors:** Mehmet Şerif AYDIN, Esra Nur YİĞİT

**Affiliations:** Regenerative and Restorative Medicine Research Center (REMER), Research Institute for Health Sciences and Technologies (SABITA), İstanbul Medipol University, İstanbul, Turkiye

**Keywords:** Dorsal root ganglion, intraganglionic injection, intrathecal injection, multiphoton microscopy

## Abstract

**Background/aim:**

Dorsal root ganglia (DRG) are structures containing primary sensory neurons. Intraganglionic (IG) and intrathecal (IT) applications are the most common methods used for viral vector transfer to DRG. We aim to compare the efficiencies and pathological effects of IT and IG viral vector delivery methods to DRG, through in vivo imaging.

**Materials and methods:**

Mice were divided into four groups of six each: IT, IG, IT-vehicle, and IG-vehicle. Adeno-associated virus (AAV) injection was performed for EGFP expression in IT/IG groups. DRGs were made visible through vertebral window surgery and visualized with multiphoton microscopy. After imaging, spinal cords and DRGs were removed and cleared, then imaged with light sheet microscopy.

**Results:**

No neuronal death was observed after IT injection, while the death rate was 17% 24 h after IG injection. EGFP expression efficiencies were 90%–95% of neurons in both groups. EGFP expression was only observed in targeted L2 DRG after IG injection, while it was observed in DRGs located between L1–L5 levels after IT injection.

**Conclusion:**

IT injection is a more suitable method for labeling DRG neurons in neurodegenerative injury models. However, when the innervation of DRG needs to be specifically studied, IT injection reduces this specificity due to its spread. In these studies, IG injection is the most suitable method for labeling single DRG neurons.

## 1. Introduction

The peripheral nervous system (PNS) exchanges motor, sensory, and autonomic information between the central nervous system (CNS) and effector organs and tissues. One of the anatomically well-defined structures in the PNS is the dorsal root ganglia (DRG), which contains primary sensory neurons. DRG neurons emerge from the dorsal root of spinal nerves and carry sensory information from various receptors, including pain and temperature, to the CNS [1]. DRG has significant clinical implications, particularly in the context of neuropathic pain, making it a significant target for injection applications of new therapies aimed at treating chronic pain. Current pharmacological agents used to treat chronic pain primarily act through nonspecific analgesic mechanisms on the intrinsic pathways of pain and tend to cause side effects [2]. Therefore, understanding the complex cellular mechanisms and pathways that contribute to pain will facilitate the development of new mechanism-based pain management therapies [3]. Chronic pain is a common subject of preclinical studies, and one of the objectives is to develop useful methods, such as intraganglionic injection (IG), to target DRG with analgesics [4]. However, accessing the DRG surgically is difficult due to the bony confines of the intervertebral foramina. Nevertheless, this difficulty can be overcome using spinal surgery in preclinical studies [5, 6].

Gene transfer to primary sensory neurons in the nociceptive pathway is a current approach to investigating pain. Gene transfer to nociceptors is also a promising strategy for managing chronic pain, as it allows selective targeting of pain pathways without producing off-target effects by enabling the expression of a transgene in limited regions of the nervous system [3]. Due to containing the cell bodies of sensory neurons, DRGs are the optimal anatomical target for examining sensory axon regeneration and applying viral vectors in gene therapy. Recently, several serotypes of adeno-associated virus (AAV) have been reported to effectively transfer genes to DRG neurons in rodents. However, the efficiency of gene transfer in DRG varies significantly depending on the delivery methods, animal species, and viral serotypes [3, 7, 8].

Different strategies have been tried for gene transfer to DRG neurons, including liposomal, lentiviral, cationic polymers, and electroporation [9–12]. The most common methods used for viral vector transfer into the DRG are direct IG injection [5, 8, 13–15] or intrathecal (IT) application into the cerebrospinal fluid [7, 16–20]. Studies in which AAVs were applied at high doses of IT, IG, or systemically reported no damage to the DRG [5, 14, 21], but pathological damage or toxicity has also been shown to occur [22, 23]. IG injection using glass micropipettes causes minimal trauma to the DRG and is effective only within the DRG without broad passage to spinal roots [5]. One of the greatest advantages of this technique is that it minimizes the amount of injected chemicals to affect only the cell bodies of DRG neurons, single spinal afferent axons, and their associated nerve endings [24]. AAVs directly injected into rat DRGs achieve gene transfer to up to 90% of neurons, including most nociceptors [13] . The approach of delivering AAVs directly to DRGs is an effective method for producing expression only in DRG neurons, allowing targeted visualization of these neurons and their associated afferent nerves without confusion from other axons in areas of interest.

IT injection performed easily through lumbar puncture is an alternative injection site for DRG. However, a relatively high volume of the agent is required compared to IG injection, and this route allows the applied agent to be effective at multiple segmental levels bilaterally [25]. IT injection enables the use of AAVs that affect DRG neurons in nociception and neuropathic disease processes [26]. Following IT AAV injection in mice, it was shown that large-sized DRG neurons are affected while nonpeptidergic nociceptors are not affected [12] .

Understanding the effectiveness of the injection route is important for studies aiming to target specific subsets of DRG neurons. To this end, our study addressed the effectiveness and pathological effects of IT and IG injections using in vivo DRG imaging methods to label DRG neurons with viral vectors.

## 2. Materials and methods

### 2.1. Experimental design

All animal experiments were approved by **İ**stanbul Medipol University Animal Experiments Local Ethics Committee (IMU HADYEK, approval number (604.01.01-E.46260).

6–8-week-old transgenic male mice expressing EGFP in all neurons (STOCK Mapttm1(EGFP)Klt/J) were obtained from The Jackson Laboratory (USA, #004779) and used as a positive control for in vivo DRG (dorsal root ganglion) imaging and surgical operations. 6–8-week-old wild-type C57BL/6J male mice were divided into four groups - IT (n = 6), IT-Vehicle (n = 6), IG (n = 6), and IG-Vehicle (n = 6) - and used for AAV (adeno-associated virus) and vehicle solution injection procedures. Fifty μL (1mg/mL) propidium iodide (Sigma Aldrich, P4864) was injected into the tail vein of mice at 0 and 24 h after the injections, and dead cells were identified using in vivo 2-photon microscope imaging. The same DRGs were imaged to determine the gene expression levels after 15 days of IT or IG AAV injections.

### 2.1. Vertebral window surgery procedure

Construction of the imaging window over lumbar DRGs was done as previously described [27, 28]. Mice were anesthetized with 2% isoflurane (30% O_2_) and placed onto a heating pad. The surgical incision was made in the dorsal skin at the level of L1–L4 vertebrae, paraspinal muscles, and ligaments were carefully dissected to expose DRG. The mice were then placed onto a vertebral holder to access L2 DRG for in vivo imaging. Silicon adhesive Kwik-Sil^®^ (World Precision Instruments) was applied onto DRG and covered with 3-mm diameter round cover glass. The cover glass was fixed to bone structures using cyanoacrylate adhesive (Pattex 1865943, Henkel). The vertebral window was used for in vivo DRG imaging using a 2-photon microscope.

### 2.2. Intrathecal and intraganglionic injections

The IT injections were performed as previously described [29]. Briefly, a Hamilton injector with a 32-gauge needle (Hamilton 80314, Hamilton, Reno, NV) was inserted through the L2–L3 vertebrae into the subarachnoid space during the spinal window surgical procedure. The entry into the IT space was confirmed by withdrawing a small amount of cerebrospinal fluid and 5 μL AAV-containing solution was injected.

The IG injection procedure was performed after exposure of the DRG during the spinal window surgery. A glass pipette was inserted under the DRG capsule parallel to the surface and 0.2 μL AAV-containing solution was injected. Injection procedures were performed similarly in vehicle groups using the same solutions without AAV.

### 2.4. In vivo DRG imaging through vertebral window

Through the implanted vertebral window, we performed in vivo two-photon imaging of the DRG using a vertebral holder [30]. Mice under 2% isoflurane (30% O_2_) anesthesia were imaged using a Zeiss 7 MP microscope (Carl Zeiss, Jena, Germany) equipped with dual Ti:Sapphire multiphoton lasers (Coherent Chameleon Vision II and Ultra, Coherent, Santa Clara, CA), 10x/0.45NA Plan-Apochromat objective with objective heater. 820 nm excitation laser was used to detect GFP, PI, and second harmonic generation signals of collagen fibers. Image sizes were 512 × 512 pixels, and 400–600μm thick image stacks were acquired with a z-step size of 1 μm, extending from the surface of DRG. Emission filters BP 525/50 and BP 605/70 were used to detect GFP and PI, respectively. The second harmonic generation signal of collagen fibers was detected using a BP 445/50 emission filter. All images were collected simultaneously using three different nondescanned detectors.

### 2.5. AAV preparation

High-titer AAV production was performed as described previously [31]. Briefly, AAV293 cell line was maintained in high glucose DMEM supplemented with 10% FBS and 1% NEAA and was transfected with pAAV-hSyn-EGFP, pAdDeltaF6, and pAAV2/1 (a gift from Bryan Roth, and gifts from James M. Wilson, plasmids were purchased from Addgene #50465, #112867, #101276). Cells were collected 72h post-transfection and lysed by 3x freeze-thaw cycles in lysis buffer followed by sonication. RNA and DNA impurities were removed by Benzonase (Sigma Aldrich). Cell debris was removed by centrifugation and the supernatant is ultracentrifuged through iodixanol gradient (17%, 25%, 40%, 60%). AAV is collected from 40% fraction. AAV particles were concentrated in storage buffer (1 × PBS containing 5% D-sorbitol) using Amicon 100K columns (Sigma Aldrich and stored at −80 °C for further usage.

### 2.6. Tissue clearing and whole-tissue imaging

Tissue clearing was performed by using the combined and modified versions of CUBIC and uDISCO protocols [32, 33]. Mice were anesthetized with 1.5%–2.0% isoflurane and fixed by intracardiac perfusion of heparinized PBS and 4% PFA in PBS. The spinal cord segment containing thoracic and lumbar DRGs was removed from sacrificed mice. All harvested samples were postfixed overnight at 4 °C in 4% PFA in PBS. The next day, the tissues were washed in PBS for 1 h. For delipidation and decolorization, the tissues were placed in 1:1 dH2O:CUBIC-L and CUBIC-L (10% N-Butyldiethanolamine (Sigma 471240), 10% Triton X-100 (Sigma X100), 80% dH2O) solutions and incubated at 37 °C for 1 day each. The tissues were dehydrated in a graded series of tert-butanol (Merck 82264) in PBS (30%, 50%, 70%, 80%, 90%, 96%, 100%, and 100%, 3 h each). Tissues were immersed in BABB-D10 solution (10:20:3; Benzyl-alcohol (Merck 100981), benzyl benzoate (Merck 818701), and Di-Phenyl ether (Thermoscientific A15791)) for refractive index matching. Cleared tissues were imaged using a Light sheet microscope (Lightsheet 7, Carl Zeiss, Jena, Germany) with a 5x/0.16NA objective and 488nm excitation laser in the BABB-D10 solution.

### 2.7. Statistical analysis

Student’s t-test was performed using GraphPad Prism version 9.0.0 for Windows, (GraphPad Software, San Diego, California, USA). Throughout the study, p-values< 0.05 were considered statistically significant. Error bars in graphs represented as SEM.

## 3. Results

**Figure 1:**
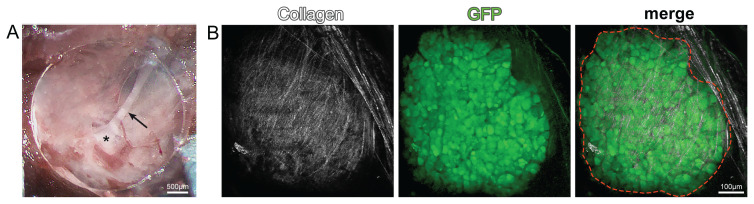
Vertebral window surgery and multiphoton image of DRG. (A) The macroscopic image of the DRG (*) and peripheral process (arrow) covered with glass after vertebral window surgery. (B) In vivo multiphoton images of DRG from Mapttm1(EGFP)Klt/J mice. Collagen surrounding the DRG in a capsule (white), neurons expressing GFP within the DRG (green), and the anatomical boundaries of the DRG (red dashed lines).

A stable vertebral imaging system for long-term in vivo imaging was achieved by using a clear silicon adhesive and round cover glass (Figure 1A). EGFP- expressing DRG neurons were clearly seen through the vertebral window (Figure 1B).

**Figure 2:**
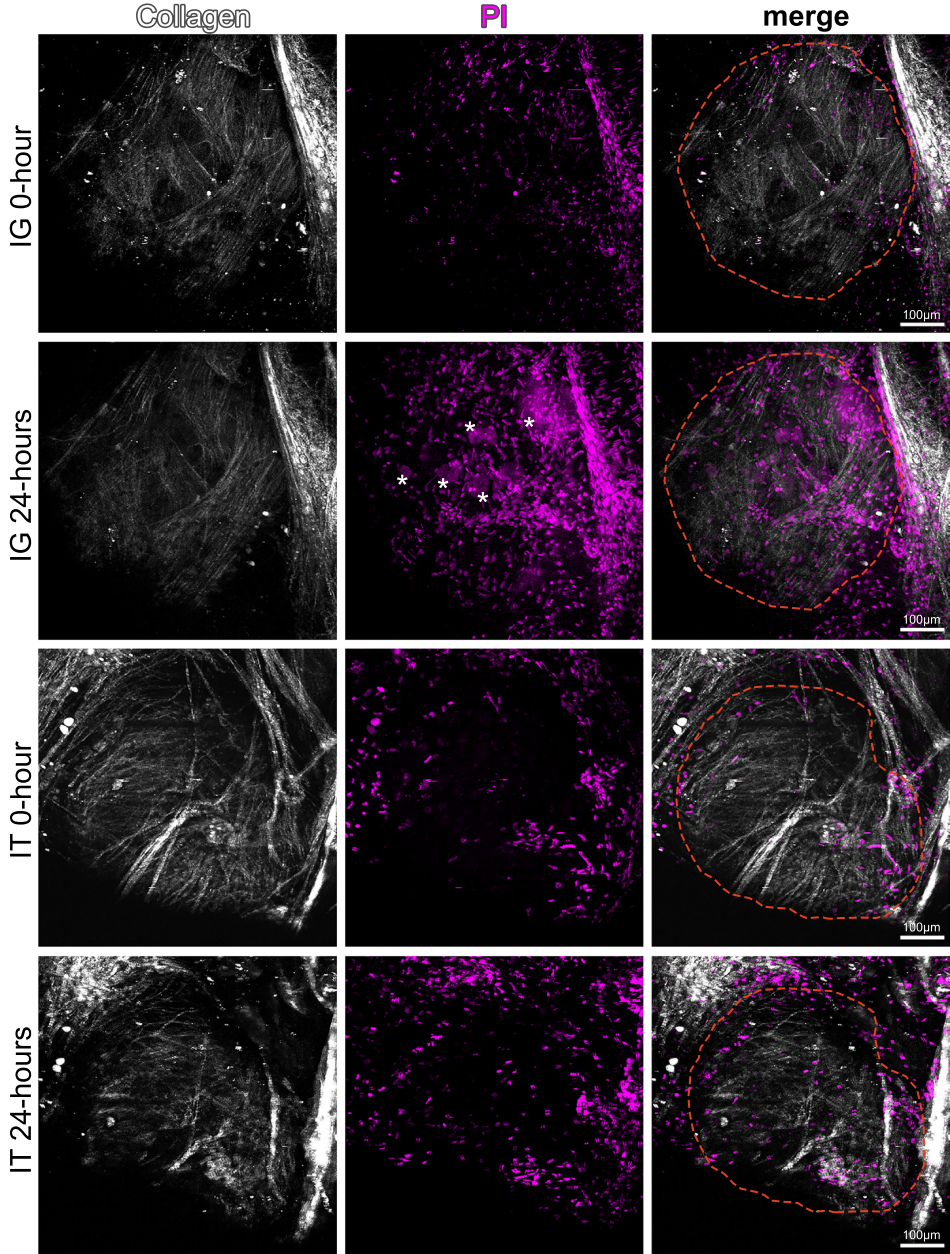
In vivo DRG imaging of cell death in mice after injections. Collagen in the capsule surrounding the DRG (white), dead cells stained with PI (magenta), and the anatomical boundaries of the DRG (red dashed lines). During surgical exposure of the DRG, a small amount of cell death was observed in the connective tissue cells in the capsule (magenta, small flat nuclei) in all groups. Dead neurons (*, large round nuclei) in the DRG after 24 h of IG injection.

To determine the pathological effects of the injection method on DRG, in vivo imaging was performed at 0 and 24 h after IT and IG injections. No changes in DRG morphology or neuronal death were observed in the 0-h imaging after IG injection, while microscopic examination conducted 24 h after injection revealed 17.33% ± 9.04% cell death in neurons in the same region. In microscopic examinations performed at 0 and 24 h after IT injection, no morphological damage or cell death was observed in DRGs at the level of the injected vertebra (Figure 2).

**Figure 3:**
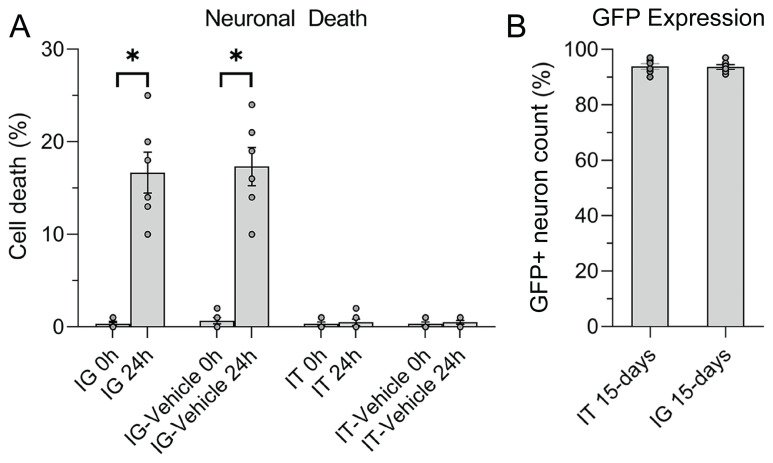
Cell death and transduction efficiency analysis of DRG neurons. (A) Time-dependent neuron death in the DRG following IT and IG injections and (B) GFP expression in DRG neurons 15 days after IT and IG AAV injections.

To determine if the cell death observed in the injection was due to AAV or transgene, IG-vehicle and IT-vehicle injections were performed. Microscopic examination revealed no visible cell death in the DRG at 0 h after injections, while 16.67% ± 5.42%, 17.33% ± 5.04%, 0.33% ± 0.51% and 0.50% ± 0.83% cell death was detected at 24 h after IG, IG-vehicle, IT, and IT-vehicle injections, respectively (Figure 3A).

**Figure 4:**
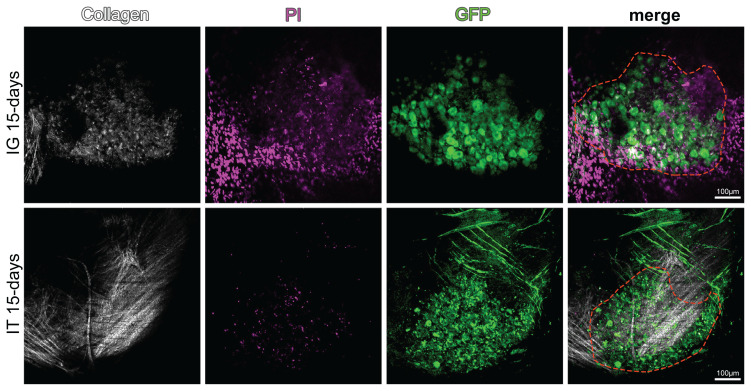
In vivo imaging of GFP+ DRG neurons after injections. In vivo microscopic examinations performed 15 days after IG and IT injections of AAV. Collagen (white) surrounding the DRG capsule, dead cells (magenta), GFP expressing neurons (green), and the anatomical boundaries of the DRG (red dashed lines).

To determine the spread and efficiency of AAV depending on the injection method, IT and IG AAV injections were performed. Fifteen days waiting period was required for AAV-dependent gene expression to occur after injection. At the end of 15 days, the DRG in the injected area was imaged in vivo with a multiphoton microscope. In IG injections, 94.0% ± 2.91% GFP expression was observed in live neurons within the DRG. Following IT injection, GFP expression was determined 93.80% ± 2.38% of neurons. There was no statistically significant difference in GFP-expressing neuron numbers between IT and IG injections (Figure 3B and Figure 4).

**Figure 5:**
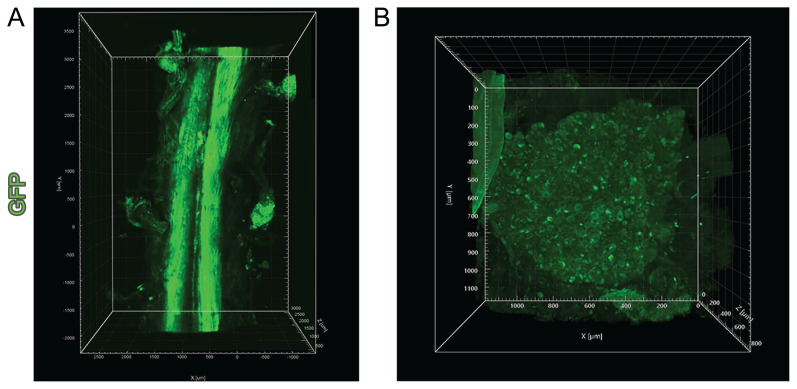
Light sheet image of GFP+ neurons after IT injection. (A) AAV spread in spinal cord samples taken out 15 days after AAV injection. GFP expression was observed not only in the injected DRG level but also in the axons extending throughout the spinal cord and in the DRGs in the lower and upper segments. (B) High magnification image of a single DRG containing GFP+ neurons after injection.

To determine the area affected by the injection and the extent of its spread, the DRGs and spinal cord were removed from the sacrificed mice 15 days after injection following in vivo imaging. Tissue clearing was applied to enable microscopic imaging of the entire tissue. The spinal cord segment related to the injection site was imaged using a light sheet microscope. After IG injection, GFP expression was only observed in the injected DRG. Following IT injection at the L3 vertebral level, GFP expression was observed in DRGs at the L1-L5 vertebral levels depending on the cranial and caudal spread in the cerebrospinal fluid (Figure 5).

## 4. Discussion

The administration of viral vectors such as AAVs has been a promising approach in gene therapy for various neurological disorders. To compare the routes of administration of AAV to DRG, we investigated the efficiency and effects of AAV following IG and IT injections. Our research aimed to investigate the impact of injection methods on DRG neurons. No neuronal death was observed after IT injection, whereas there was a significant amount of neuron death following IG injection. Similar to the IG injection, cell death observed in IG-vehicle group indicates that the effect was due to mechanical damage during invasive intervention into the DRG with a glass pipette. The lack of cell death in DRG after IT injections support this perspective. Although IG injection is widely used for viral administration to DRG in many studies, a few investigated the pathological outcomes of the surgical procedure itself. Various studies report effects such as satellite activation [34], increased Iba1 and ATF3 expression [35] after IG injection. Nevertheless, there are studies stating that these outcomes of IG injection are transient and tolerable hence can be used as a therapeutic administration route [8, 36, 37]. To minimize the mechanical side effects of IG injection, the injection rate and the amount of solution can be reduced [5], still the surgery must be done by an experienced and well-trained researcher.

When we compared the effects of AAV on gene expression in DRG neurons, we observed similar numbers of GFP-expressing neurons following IG and IT injections. This indicates that both injection methods are successful in terms of transduction efficiency. There are several reports holding different results regarding the IT and IG injection. AAV serotype, concentration and injection volume are possibly the main factors determining these differences. A previous study using different AAV serotypes stated that using AAV2 and AAV6 are not able to produce transgene expression in DRG [38]. In contrast, Towne et al. [3] showed that AAV6 injection is suitable for DRG transduction in both IT and IG injections. In our study, we produced high titer (~10^12^) and purity AAV2/1 serotype containing GFP transgene under hSyn promoter. High titer virus production enabled us to inject low amounts (5 μL for IT and 0.2 μL for IG injection) of the virus which possibly have an influence on high transduction efficiency. Consistent with our results, Iwamoto et al. [39] showed that IT injection of AAV1 with CAG promoter-driven transgene resulted with robust DRG and spinal cord expression. Similarly, Mason et al. [8] showed that AAV1, AAV5, and AAV6 are the serotypes that transduced most neurons in IG injection.

While we observed gene expression only in the targeted DRG after IG injection, expression was also seen in DRGs located in the caudal and dorsal directions after IT injection. This shows that AAV given in IG injection is limited within a single DRG, while in IT injection it spreads through the cerebrospinal fluid and affects neighboring regions. If gene expression is desired specifically in neurons, neuron using a neural promoter may be a better choice compared to the ubiquitously active promoters. Additionally, using a Cre-lox system combined with DRG-specific promoter may be another option to overcome the specificity issue in IT injection. To specifically examine a single DRG and the areas it innervates, IG injection can be used as an effective method due to its lack of spread outside of the target region. However, neuron death in the DRG due to IG injection should be carefully considered. Peripheral nerve injection is another method for targeting DRG, but this carries the risk of interfascicular injection and nerve damage [5]. In applications involving more than one DRG, IG injection becomes difficult to apply to each DRG because it requires surgical intervention. IT injection is the most practical application method when it is desired to target multiple DRGs. Depending on the targeted DRGs and their localization, the volume of AAV and the IT injection sites must be carefully considered.

In conclusion, our findings have important implications for gene therapy applications targeting DRG. The choice of injection site and route of administration can significantly affect the spread of the therapeutic agent and its effectiveness. IG injection may be more appropriate for targeting a specific DRG, while IT injection may be better suited for a more widespread distribution of the agent. The use of different viral vectors and doses may also affect the spread and efficacy. The findings may guide the application of more effective and targeted gene therapy approaches for DRG-related applications.
